# Improvements in Temperature Uniformity in Carbon Fiber Composites during Microwave-Curing Processes via a Recently Developed Microwave Equipped with a Three-Dimensional Motion System

**DOI:** 10.3390/ma16020705

**Published:** 2023-01-11

**Authors:** Kaihua Chen, Guozhen Zhao, Jing Chen, Xiaobao Zhu, Shenghui Guo

**Affiliations:** 1 Faculty of Metallurgical and Energy Engineering, Kunming University of Science and Technology, Kunming 650093, China; 2 State Key Laboratory of Complex Nonferrous Metal Resources Clean Utilization, Kunming University of Science and Technology, Kunming 650093, China; 3 Key Laboratory of Unconventional Metallurgy, Ministry of Education, Kunming 650093, China

**Keywords:** carbon fiber epoxy composite, microwave curing, three-dimensional motion, temperature deviation, mechanical properties

## Abstract

Curing processes for carbon-fiber-reinforced polymer composites via microwave heating are promising alternatives to conventional thermal curing because this technology results in nonhomogeneous temperature distributions, which hinder its further development in industries. This paper proposes a novel method for improving heating homogeneities by employing three-dimensional motion with respect to the prepreg laminate used in the microwave field by using a recently developed microwave system. The maximum temperature deviation on the surface of the laminate can be controlled within 8.7 °C during the entire curing process, and it produces an average heating rate of 1.42 °C/min. The FT−IR analyses indicate that microwave heating would slightly influence hydroxyl and methylene contents in the cured laminate. The DMA measurements demonstrate that the glass transition temperatures can be improved by applying proper microwave-curing processes. Optical microscopy and mechanical tests reveal that curing the prepreg laminate by using a multistep curing process that initially cures the laminate at the resin’s lowest viscosity for 10 min followed by curing the laminate at a high temperature for a short period of time would be favorable for yielding a sample with low void contents and the desired mechanical properties. All these analyses are supposed to prove the feasibility of controlling the temperature difference during microwave-curing processes within a reasonable range and provide a cured laminate with improved properties compared with conventional thermally cured products.

## 1. Introduction

Carbon-fiber-reinforced polymer (CFRP) composites with a high strength-to-weight ratio, stiffness-to-weight ratio, and corrosion resistance have gained substantial interest in numerous fields, such as aerospace, automobiles, sporting goods [1], etc. In the automotive industry, CFRP is one of the most promising alternatives to engineered metals for lightweight automotive structural components [2,3,4,5], and its demand has increased with the booming development of electric vehicles (EV) in recent years since lightweight designs comprise effective methods for optimizing the mileage of EV [6,7]. However, due to the low through-thickness thermal conductivity of CFRP [8], as well as the demand for homogeneous temperature distributions during the entire curing process for producing the desired part quality, the curing process of the CFRP normally features long processing times and high energy consumption, especially when this process is carried out in autoclaves. Unfortunately, autoclave curing is one of the most popular curing technologies used in the current industry, and the lengthy and energy-intensive autoclave curing process is one of the primary factors for CFRP’s high production cost [9,10,11]. In order to lower the production cost of CFRPs and to expand their utilization in the modern society, energy-efficient and reliable curing technologies are currently desired for the CFRP industry.

Microwave curing is one of the promising alternatives for autoclave curing, and it features quick heating rates, high energy efficiency, and volumetric heating operations [12,13]. Published research studies report the hyperactive nature of CFRP relative to microwave-curing processes and the improved interfacial bonding of the microwave-cured part because of the selective heating operations of carbon fiber when microwave curing impinges on the prepreg, leading to improved curing at the fiber–resin interface [14,15]. Moreover, some papers also proposed combined microwave-thermal processes to further improve the part’s quality [16,17]. Although numerous papers indicated that microwave curing features high heating rates and high energy efficiency, this technology has not taken off in industries due to several drawbacks, such as inhomogeneous temperature distributions, arcing at the tips of fiber bundles, and limited penetration depths [18]. Among these issues, inhomogeneous heating might be the most unacceptable process since inhomogeneous heating would lead to inhomogeneous curing, resulting in the deterioration of mechanical strength and material consistency [19].

Non-uniform heating is related to the nature of the interaction between microwave curing and the heated material [20]. The temperature field of the heated material is primarily influenced by the internal microwave field distribution, and normally, the hot spot corresponds to the wave loop and cold spot corresponds to the wave node. However, oscillations are natural in microwave curing, and attenuation occurs when microwave distributions are transmitted in the lossy media, which would make the internal microwave field distribution more complicated. To improve microwave-heating uniformity, numerous researchers have proposed various approaches. For example, Lambert et al. [21] studied the influence of cavity designs (shape, size, microwave transmission system, etc.) on microwave field distributions inside the microwave chamber, and they indicated that hexagonal shapes could generate the most homogeneous microwave fields compared to cylindrical and rectangular shapes and other shapes. They subsequently built a HEPHISTOS microwave system, which features a hexagonal chamber and dedicatedly designed waveguides, and then they cured the unidirectional laminate by using this system to prove its capability [18,22,23]. In 2018, Daniel et al. improved this system by increasing the number of microwave sources and adding mode stirrers and a water pipe to absorb excessive microwave power [24,25]. They used this improved system to cure glass-fiber-reinforced composites and yielded a maximum temperature difference of 30 °C.

Moreover, a group of researchers from China developed two polygonal-shape microwave-heating systems. They reported an octagonal shape system in 2014, which features 16 slot antennas to transmit microwave fields into the chamber, and it was believed that this design could generate uniform microwave field distributions within the chamber [26]. Based on this system, they developed a neural network system to control the on–off state of the microwave source and the online power output to produce a compensatory heating pattern so that the temperature homogeneity increased with respect to the laminate [27]. They yielded 13.5 °C as the best temperature difference by using this system and controlling method. In 2017, they reported another heptagonal chamber equipped with 21 rectangular waveguides, and they also studied the neural system to adjust the compensatory-heating-pattern controlling method to improve temperature homogeneities [28].

Another group of researchers from China introduced microwave power into an autoclave and developed a high-pressure microwave-heating system wherein the pressure could reach up to 1.0 MPa [29]. They also adopted slot antennas to transmit microwave power in order to improve the uniformity of field distributions, but they primarily focused on the effect of curing pressures on the part’s quality rather than improving heating homogeneity [30]. Additionally, Felix et al. proposed variable frequency microwave radiation technologies to cure adhesive bonding on the glass as well as carbon-fiber-reinforced composites [31]. They demonstrated that resonating a variable-frequency microwave in the cavity could generate more homogeneous electromagnetic field distributions than that of fixed-frequency microwave fields. Although numerous technologies have been proposed to improve the heating homogeneity of carbon fiber composites in the microwave field, a reliable and easily controlled methodology is still desired for the industry. This paper introduces a novel microwave heating system equipped with a three-dimensional motion system to homogeneously heat the target material, and the unidirectional CFRP is cured by using this microwave system with the aid of vacuum bag molding. This microwave-curing methodology could yield a relatively homogeneous temperature distribution in the unidirectional laminate and control the heating rate within a reasonable value. The FT−IR was employed to study the molecular changes after the curing process. DMA, optical microscopy, tensile tests, and compressive tests were carried out to compare the quality of microwave-cured samples with that of the oven-cured samples.

## 2. Materials and Methods

### 2.1. Materials

The unidirectional carbon fiber/epoxy prepreg employed in this study was supplied by SINOPEC Shanghai Research Institute of Petrochemical Technology with a single-layered thickness of 0.2 mm. The reinforcements were continuous T300-grade carbon fibers with a mass fraction of 60 wt.%. The matrix primarily comprised epoxy and dicyandiamide (DICY), while the chemical composition of the prepreg resin was not disclosed by the manufacturers. The uncured prepreg was stored at −18 °C before use. Other consumable materials used in the experiment were purchased from Airtech.

### 2.2. Equipment

#### 2.2.1. Conventional Heating Oven

Conventional thermal curing processes were carried out in an air-circulating oven (DHG-9145A, Shanghai bluepard instruments Co., Ltd., Shanghai, China), wherein the heating rate was fixed at 5 °C/min, and the temperature could reach 300 °C.

#### 2.2.2. Microwave System

**Figure 1:**
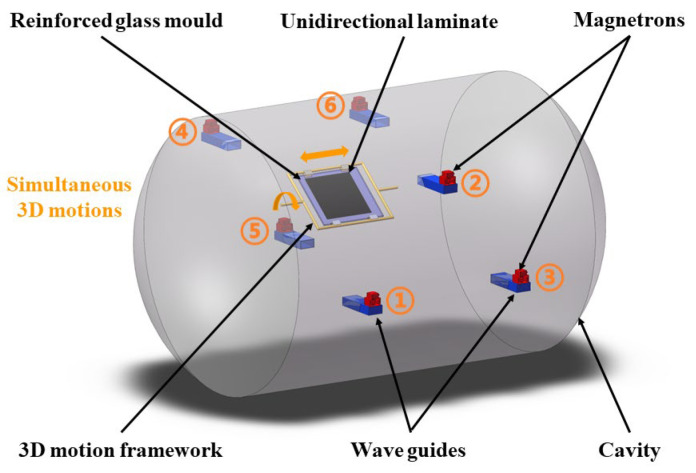
Schematic diagram of the recently developed microwave oven and composite plate used in the experiment.

A novel microwave-curing system was recently developed by the Key Laboratory of Unconventional Metallurgy, Kunming University of Science and Technology, China. As illustrated in Figure 1, this microwave equipment possesses a cylindrical configuration with two convex ends. The internal diameter of the cylinder is 1.6 m, and the total length of the chamber is 2.8 m. Six microwave inlets (rectangular waveguides) are asymmetrically distributed on two sides of the cavity to avoid the neutralization of the microwave power from different sources. Each waveguide is connected with a magnetron with 1.5 kW of power at a fixed frequency of 2.45 GHz. The power ranges from 1.11% (i.e., 100 W) to 100%, with a minimum resolution of 0.11%.

The large-sized cavity is capable of oscillating more resonant modes, which is supposed to increase the possibility of compensation among different resonant modes so that the homogeneity of field distributions can be improved. On the other hand, the three-dimensional motion framework equipped within this oven is dedicatedly designed to enable the 3D motion of laminate in microwave fields. The reciprocating linear movement as well as progressive rotation of the sample could be simultaneously realized by two motors. The temperature of the laminate is monitored by a fiber optical fluorescence measurement system, which has six fiber probes.

### 2.3. Curing Procedure

#### 2.3.1. Conventional Oven Curing

**Figure 2:**
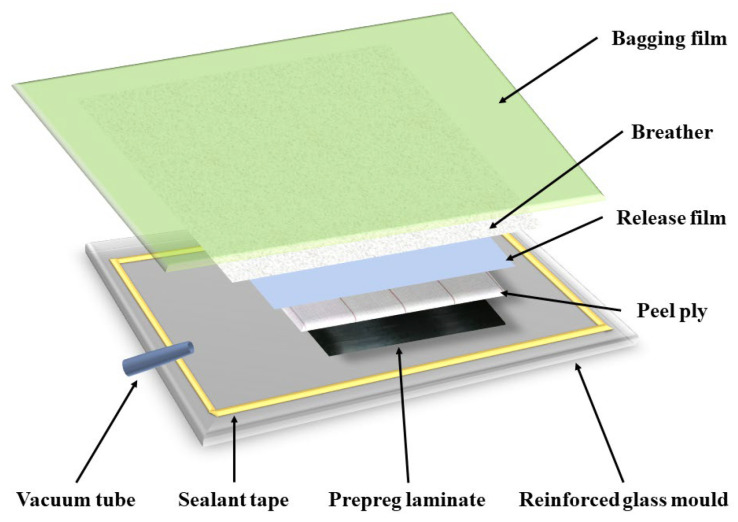
Schematic diagram of the recently developed microwave oven and composite plate used in the experiment.

The conventional curing process is supposed to be a reference: A unidirectional composite laminate consisting of 10 plies with a dimension of 300 × 300 mm was prepared by using hand lay-up methods, achieving a thickness of approximately 2.0 mm. As shown in Figure 2, typical consumables employed for oven-curing composites were used; for example, peel ply, release films, breather and bagging films, and reinforced glass (dimensions: 450 × 350 × 8 mm) were used in the mold. After finalizing the vacuum bag lay-up process of the prepreg laminate, the laminate was debulked for 30 min to remove entrapped air, and this was followed by using the supplier’s recommended cure cycle, which includes isothermally curing the prepreg at 120 °C for 120 min. Moreover, in order to reach the isothermal curing temperature, a heating rate of 5 °C/min was employed. A vacuum pressure of approximately 0.1 MPa (an atmospheric pressure) was continuously applied during the curing process.

#### 2.3.2. Microwave Curing

**Figure 3:**
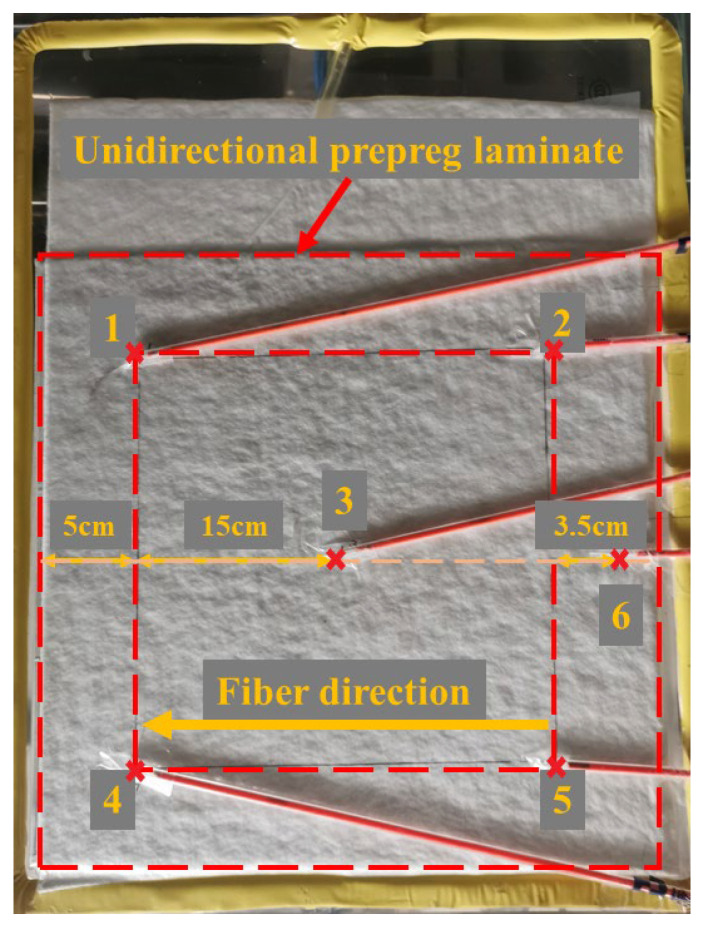
Photograph of the distribution of the fiber optical fluorescence probes on the bagging film. (The probes are numbered from 1 to 6, and their locations are corresponding to six red crosses as illustrated in this figure).

For microwave-curing processes, the same laminate lay-up sequence and geometries were employed, i.e., 300 × 300 mm [0°]_10_. The vacuum bagging arrangement was in line with that of conventional thermal curing processes. However, there were three major differences compared with conventional curing: 1. Aluminum foils extending approximately 5 mm from the edges were wrapped around each edge of the carbon fiber epoxy laminate to minimize the possibility of arcing at the tips of the carbon fiber bundle. 2. The fiber orientation should be parallel relative to the 350 mm side of the mold, which is unnecessary in conventional curing. 3. Microwave-transparent refractory processes were used to completely cover the mold and the prepreg laminate so that there was a decrease in heat dissipation from the laminate to the rest of the chamber, which was also beneficial for homogeneous temperature distribution. Additionally, the fiber probes were placed on the outer surface of the bagging film while underneath the refractory. The positions of the laminate and fiber probes are illustrated in Figure 3.

After the above processing steps, the mold was mounted on the 3D motion framework and the prepreg laminate was de-bulked for 30 min to remove any entrapped air. The microwave was subsequently switched on to heat the laminate, and then the reciprocating linear movement and progressive rotation were, respectively, switched on at 5 s and 10 s. Moreover, only four magnetrons (No. 2, 3, 4, and 6) were simultaneously switched on during the entire curing process, and the output of each magnetron was the same. In order to retain a reasonable heating rate, the microwave’s power level increased step-by-step during the curing process. Additionally, reciprocating linear movements were carried out within a range of 30 cm, the speed was fixed at 1 cm/s, and the time interval between each round of reciprocating motion was 5 s. Progressive rotations were initialized from the vertical position at a constant speed of 2.5 rpm, while the time interval between each round of progressive rotation was 3 s. The 3D motions of the laminate were continuously carried out until the end of the isothermal curing segment.

Considering the dynamic rheometry of the prepreg obtained at a constant heating rate of 1 °C/min (Figure S1) and the isothermal DSC results obtained at 120 °C and 140 °C (Figure S2), a series of cure cycles (Table 1) was defined for the microwave processing operations of carbon fiber/epoxy composite laminates. Isothermal curing processes at 110 °C were carried out for 10 min in M2 and M4 cycles, and these processes were supposed to improve resin flow and fiber-surface-wetting operations since the prepreg’s viscosity was the lowest at 110 °C. During the isothermal curing process, the microwave power was intermittently switched on to maintain the temperature. After the finalization of the curing process, the microwave power was switched off, and the refractory was immediately removed to allow the laminate to naturally cool down.

### 2.4. Characterization of the Cured Laminates

#### 2.4.1. FT−IR

Fourier-transform infrared (FT−IR) measurements were conducted using the Bruker Tensor 27 spectrometer at room temperature. The uncured prepreg was measured by using ATP technology, and the wave number ranged from 600 cm^−1^ to 4000 cm^−1^. The cured sample was tested by using KBr pellets, and the wave number ranged from 400 cm^−1^ to 4000 cm^−1^.

#### 2.4.2. Dynamic Mechanical Analysis for Glass Transition Temperatures

The glass transition temperatures of various cured laminates were determined according to ASTM Standard D7028-2007 to test the curing degrees of various cured laminates. The measurements were performed on a TA DMA 850 machine using a three-point flexural loading mode at a fixed frequency of 1 Hz and a fixed heating rate of 5 °C/min. The specimen was cut from the cured laminate. The coupon size was 60 × 11 mm, with the fiber direction oriented along the length axis of the specimen, and the thickness varied between 2 mm and 2.3 mm. For each laminate, two specimens were tested, and an average value was taken.

#### 2.4.3. Void Content

Optical microscopy and image analyses were used to determine the void content in the composites. The specimens were mounted in epoxy resin, ground, and then polished to obtain a high-quality surface. The pictures were captured and saved by using Leica DMI8 optical microscopy. For each laminate, void content measurements were performed with 20 images acquired at 20 polished surfaces, and the average values were taken.

#### 2.4.4. Mechanical Testing

The tensile strength and modulus were determined according to ASTM Standard D3039, and the test was performed using the INSTRON 5982 machine. The samples were cut from the cured laminate to a size of 250 mm in length and 15 mm in width. Aluminum tabs were used with a dimension of 56 × 15 × 1.5 mm. The test speed was 2 mm/min, five coupons from each laminate were tested, and the average value was taken.

The 0° direction compressive properties based on ASTM Standard D6641/D6641M-16 (E2017) were also conducted using the INSTRON 5982 machine. The specimens were 140 mm in length and 13 mm in width. Aluminum tabs were also used with dimensions of 63.5 × 13 × 1.5 mm. The test speed was 1.3 mm/min, five coupons from each laminate were tested, and the average value was taken.

## 3. Results and Discussion

### 3.1. Microwave Heating Characteristics

The microwave-radiation-induced arcing at the tips of the carbon fiber bundles is one of the major barriers for microwave-curing technologies. Numerous research studies have been conducted with a focus on avoiding arcing by using aluminum foils to ground the tips [11] or curing the CFRP in a sulfur hexafluoride gas atmosphere to increase the carbon fiber gaseous breakdown voltage [32]. This paper proposes another method to avoid arcing phenomena by increasing the volume of the cavity to decrease the microwave power intensity inside the chamber and, therefore, can reduce microwave absorptions at the tips of fiber bundles, which is also beneficial for homogeneously heating the CFRP. In this case, the microwave-curing operation of the composite laminates without arcing at a curing pressure of 0.1 MPa throughout the microwave processing cycle could be realized. Moreover, the decreased microwave power intensity in a large-sized chamber is beneficial for controlling a reasonable heating rate for the curing of CFRP.

**Figure 4:**
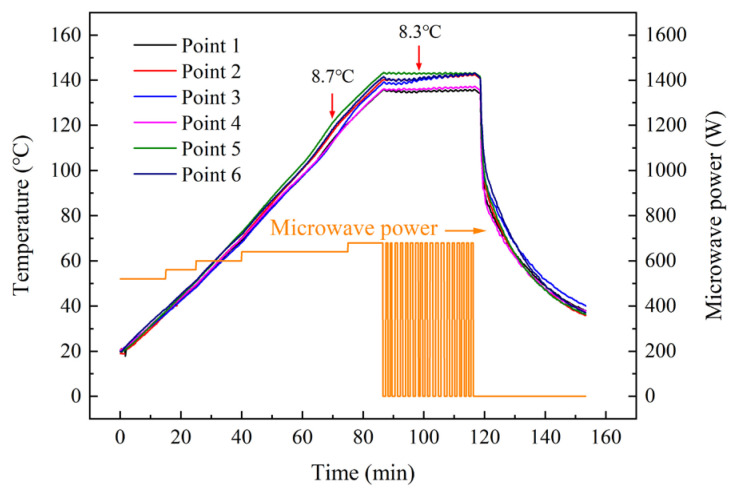
Temperature characteristics of the unidirectional laminate by microwave heating.

The temperature profiles of various microwave-curing processes demonstrate that the maximum temperature deviation ranged from 8.7 °C to 13.8 °C, and the heating curves with the best temperature difference control are shown in Figure 4. The temperature profiles of M1, M2, and M4 are presented in Figure S3. As shown in Figure 4, it can be observed that the initial temperature of the uncured laminate was 18.9~20 °C, and the deviation was caused by an instrumental error in the fiber system. During the heating process, the microwave power increased in four steps, and the power output increased by 40 W each time. In total, the microwave power ranged from 520 W to 680 W. The temperature at various points could reach 135.6~143 °C within 86.5 min, and the average heating rate was about 1.42 °C/min. After the highest temperature at six points reached 143 °C, the curing process entered the isothermal segment, which lasted for 30 min. The on/off state with respect to microwave power was controlled by the PID system so that the temperature could be maintained during the isothermal segment. However, the temperature of point 3 and point 6 soared to 143 °C after ca. 96 min, which might be attributed to the lower heating dissipation at these two regions compared with other regions.

### 3.2. Molecular Structure Analysis by FTIR

**Figure 5:**
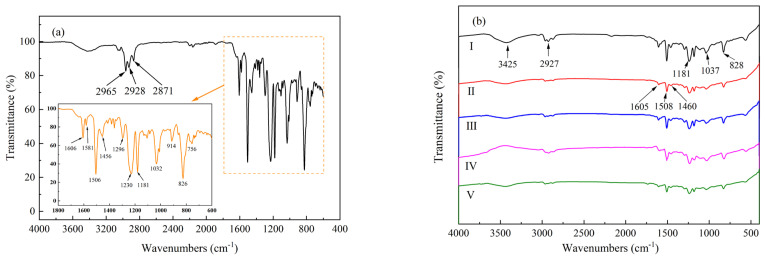
FT−IR patterns for (**a**) uncured prepreg and (**b**) laminates cured by various procedures: I conventional oven-cured sample, II M1, III M2, IV M3, and V M4.

The influence of microwave curing on the molecular structure of cured products was investigated using FTIR. Figure 5a,b illustrate the FTIR spectra of the uncured prepreg and the laminates cured by various cure cycles, respectively. As shown in Figure 5a, the absorption band at 3425 cm^−1^ is attributed to the stretching of hydroxyl groups [33]. The bands located at 2965, 2928, and 2871 cm^−1^ corresponded to the stretching of the C-H and C-H_2_ groups [34]. The peaks at 1606, 1506, and 1456 cm^−1^ are assigned relative to the C=C group of the aromatic rings [35]. The peak at 1581 cm^−1^ is the characteristic stretching vibration of N-C=N [33], and this is referred to as the DICY curing agent. The bands located at 1230 and 826 cm^−1^ were due to the C-O stretching and C-H deformation at the para position of the aromatic rings, respectively [35]. Moreover, the peak at 914 cm^−1^ corresponded to the vibration of epoxy ring [36].

The FT−IR spectrum of the cured samples (Figure 5b) presents several spectral changes. The peak at 914 cm^−1^ and 1581 cm^−1^ cannot be detected in all cured samples, suggesting the consumption of epoxide groups and DICY during the curing process. Moreover, the peak intensities of 3425 cm^−1^ and 2927 cm^−1^ are more prominent in the conventional oven-cured sample compared to microwave-cured samples. The greater intensity of the 3425 cm^−1^ peak indicates higher water content in conventional thermally cured samples, which could be attributed to the selective heating of water by using microwave power, leading to an improvement in water evaporation in microwave-curing processes. On the other hand, the peak of 2927 cm^−1^ is attributed to the symmetric stretching of the C-H band of CH_2_. The weakened peak intensities of 2927 cm^−1^ in the microwave-cured samples indicate the lower CH_2_ content compared with traditional thermally cured samples. It has been reported that the homogenous cleavage of the covalent bonds of methylene structure would occur at high temperatures, producing a relatively stable carbon radical and a highly reactive hydrogen radical. These radicals would then participate in the curing reaction of the nitrile group, and this finally leads to lower methylene contents [37]. The curing temperatures that are employed in M3 and M4 cycles were higher than the TC cycle, and the temperatures in the M1 and M2 cycles were also higher than 120 °C due to the nonuniform heating processes in the microwave field. Therefore, the reduced band intensities of 2927 cm^−1^ in the microwave-cured samples might be attributed to the higher curing temperatures, leading to the participation of methylene groups in the curing process. Nevertheless, absorbance bands at 828, 1460, 1507, and 1605 cm^−1^ were still detected, and they indicate the remaining aromatic rings.

### 3.3. Tg by DMA

**Figure 6:**
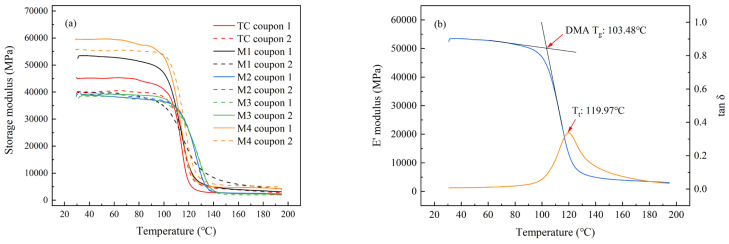
(**a**) Storage modulus versus temperature for various CFRP coupons. (**b**) Construction of the storage modulus for glass transition temperatures of M1 coupon 1 (DMA Tg: glass transition temperature; Tt: peak temperature of tangent delta curve).

DMA temperature scan tests were performed on various carbon/epoxy composite samples at a 1 Hz frequency at a heating rate of 5 °C/min. The evolution of the storage modulus with the increase in temperature is shown in Figure 6a. With an increase in temperature, the storage modulus values for all samples decreased, and the process could be divided into three stages: The first stage corresponds to the mild decrease in the E’ value with the increase in temperature, which indicates that the epoxy matrix is in a glassy state and the laminates have a high storage modulus. The second stage is the dramatic decrease in E’ value, representing the glass transition of the resin matrix. The final stage corresponds to low modulus values due to the epoxy matrix that is transferred to a rubbery state, and this increases the mobility of polymer chains as well as the energy dissipation [38]. On the other hand, the storage moduli of various samples at 30 °C are summarized in Table 2. It can be observed that the average E’ value for the TC sample at 30 °C was 42,413 MPa, and only the E’ values of two M4 coupons and M1 coupon 1 were higher than this value. However, M1 coupon 2 had a lower E’ value of 40,191 MPa, which might be attributed to the inhomogeneous curing processes in the microwave field. All E’ values of the M2 and M3 coupons were lower than that of the TC sample. Considering that the dynamic storage modulus is approximately similar to Young’s modulus and can reflect the rigidity of materials [39], it can be concluded that the M4 sample has higher rigidity than that of the TC sample, indicating that the M4 cycle could improve the stiffness of the cured laminate.

Figure 6b presents the temperature scan of the carbon fiber/epoxy composite cured by the M1 cycle, and the temperature at which a significant drop in the storage modulus (E’) begins was assigned as the glass transition temperature. The T_g_ values of various laminates obtained by microwave-curing processes are also summarized in Table 2.

According to the non-isothermal DSC scans reported in another paper [40], the average ultimate glass transition temperature (T_g,∞_) of this prepreg system is 106.2 °C. The T_g_ value of the sample cured by the TC cycle (107.5 °C) was similar to the T_g,∞_ obtained by DSC, which demonstrates that the laminate produced by the TC cycle was fully cured. However, the T_g_ values of the M1 and M3 specimens were both slightly lower than the TC sample, indicating the lower curing degree of the M1 and M3 samples. Although the TC cycle and M1 cycle both cured the prepreg laminate at 120 °C for 120 min, the M1 sample still produced a lower T_g_, which might be attributed to the inhomogeneous temperature distribution in the microwave field.

On the other hand, the M2 and M4 cycles both produced higher T_g_ values than the TC specimen. These two cycles both had an isothermal segment at prepreg’s lowest viscosity for 10 min, and this should be beneficial for resin flow and fiber-wetting processes. However, detailed studies on the higher T_g_ values compared to T_g,∞_ with respect to these two specimens are currently in process.

### 3.4. Comparison of Physical and Mechanical Properties

**Figure 7:**
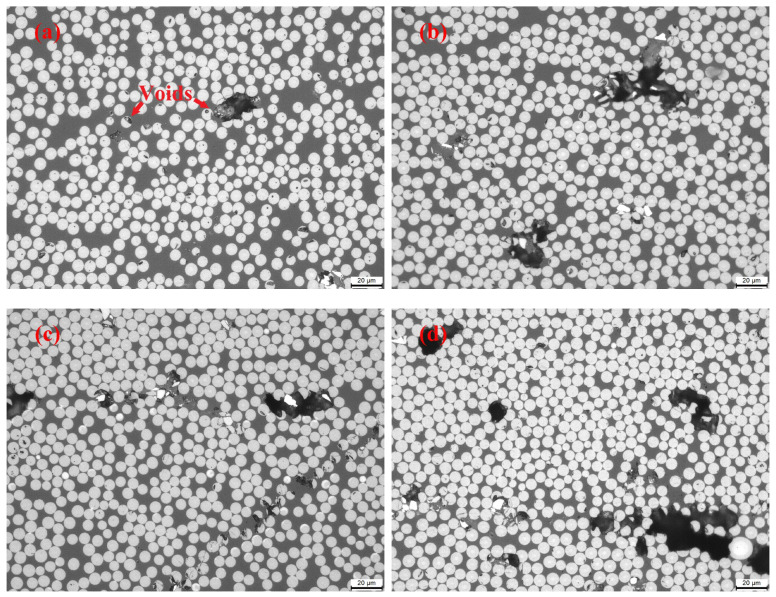

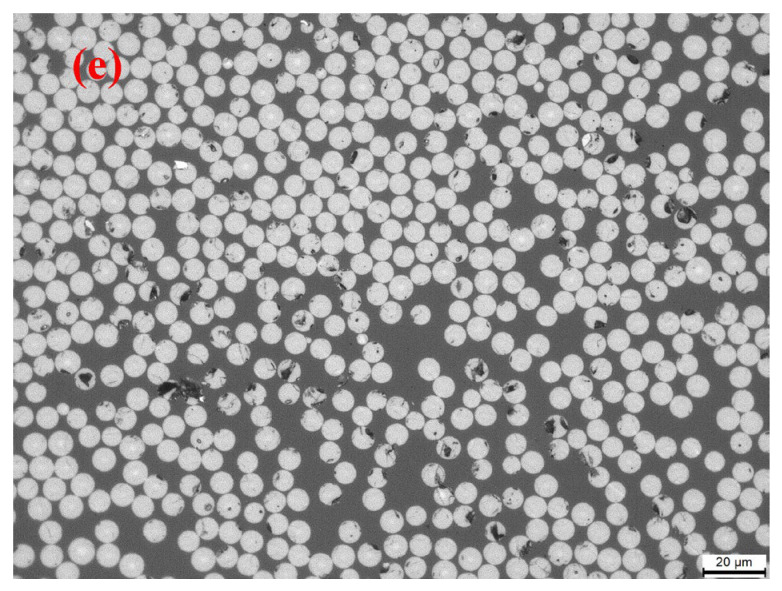
Optical micrographs of (**a**) TC, (**b**) M1, (**c**) M2, (**d**) M3, and (**e**) M4 processed laminates.

Representative optical micrographs taken at 500× magnification for conventional curing and microwave-curing processes are shown in Figure 7. By carefully inspecting these images, the void contents of the TC, M1, M2, M3, and M4 samples were 8.4%, 11.4%, 9.9%, 15.5%, and 4.7%, respectively. These photographs reveal that different microwave-heating profiles had significant influences on the void contents of the cured sample. The TC and M1 cycles both cured the prepreg laminate at 120 °C for 120 min, while the M1 specimen displayed higher void contents than the TC sample. This phenomenon was also reported by Nightingale et al. [16], and this result is not desired by manufacturers. Moreover, curing the prepreg laminate by using multistep processes can efficiently decrease the void content, since the isothermal segment at the lowest viscosity is beneficial for fiber surface wetting processes in the resin matrix. Therefore, the void content of the M2 sample was slightly lower than that of M1, and the void content of M4 was further reduced to 4.7%, which was much lower than the conventional thermally cured sample.

**Figure 8:**
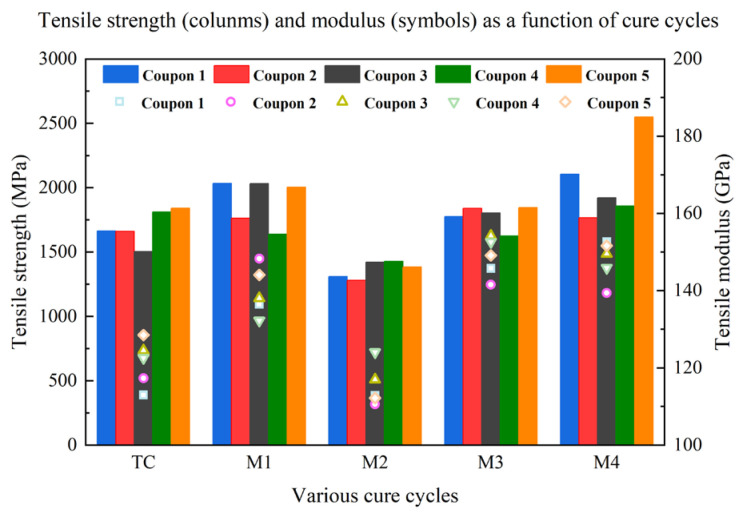
Longitudinal tensile strength and modulus of samples cured by various cure cycles.

The 0° tensile tests were carried out to investigate the load-carrying capacity of carbon fiber/epoxy composites and to evaluate the effect of various microwave-curing cycles with respect to the mechanical properties. Figure 8 presents the tensile strength and tensile modulus results for composite laminates cured using conventional thermal and microwave processes. In comparison to the thermally cured laminate, the tensile strengths of the M1, M3, and M4 samples had higher values, while M2 displayed a reduced tensile property. Exhibiting the maximum tensile strength value, the microwave-cured composite, M4, exhibited a noticeable improvement in the tensile strength, which was observed to be 20.3% higher than the conventionally cured samples. However, the 19.6% decrease in tensile strength of the M2 sample compared with the TC-cured laminate was unexpected, since the multistep curing process is supposed to improve resin–fiber adhesion by adding an isothermal stage at the lowest viscosity of the resin matrix. By taking the maximum temperature deviation into consideration, it can be observed from Figure 4 and Figure S1 that the temperature difference was the largest in the M2 cycle among all microwave-curing processes. During the M2 curing process, it can be observed that the temperature at point 5 continuously decreased in the second isothermal curing stage, which might be a result of a poor retainment of warmer temperatures. In this case, it can be concluded that a homogeneous temperature distribution during the microwave-curing process would be a significant factor that influences the mechanical properties. As for the tensile modulus, the M1, M3, and M4 samples all had higher tensile modulus values than the conventional thermally cured one, while that of M2 had a slightly lower value than the TC sample. This regularity is in agreement with that of the tensile strength value.

**Figure 9:**
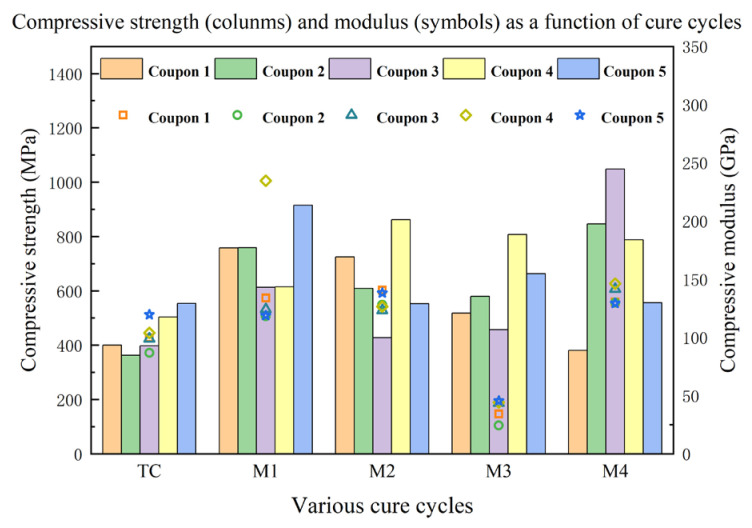
The 0° compressive strength and modulus of laminates cured by different cure cycles.

The effect of different curing processes on the 0° compressive properties is summarized in Figure 9. These results indicate that all microwave-cured laminates exhibited higher compressive strengths than the conventionally cured sample. The microwave-cured M1 composite presented the highest compressive strength of 732.07 MPa, which is a remarkable improvement (65.0%) compared with the conventional thermally cured sample of 443.72 MPa. Likewise, the compressive strength of laminates M2, M3, and M4 increased by 43.2%, 36.3%, and 63.2%, respectively. However, the compressive strengths of the M2 and M4 composite panels displayed a high degree of variation, which was perhaps caused by poor temperature uniformity during microwave radiation processes, which was not detected by the optical fiber fluorescence system. As for the compressive modulus, the M1, M2, and M4 samples were slightly higher than the thermally cured one, while that of M3 was significantly lower than the TC sample. The lower compressive modulus of the M3 sample might be attributed to the highest void content and relatively low curing degree indicated by lower glass transition temperatures.

Table 3 provides the maximum temperature deviation, void content, tensile strength and modulus, compressive strength, and the modulus for the laminates manufactured by using various methods. According to these data, it could be concluded that the temperature uniformity during the microwave-curing process and curing cycle are the two factors that significantly influence the overall properties of microwave-cured laminates. Firstly, poor temperature homogeneity during the microwave-heating process results in a large temperature deviation, which normally leads to poor mechanical properties. Moreover, the maximum temperature deviation obtained in multistep processes is usually higher than that of single-step processes, since it would be more difficult to maintain homogeneous temperatures during longer microwave-curing times. However, adding an isothermal curing stage at the resin’s lowest viscosity is beneficial for reducing void contents, which would compensate for the larger temperature difference induced by longer curing times and improve the overall mechanical properties to some extent. Therefore, short periods of curing would be favorable in the microwave field for realizing a uniform heating pattern. In this case, a high curing temperature should be employed to shorten the curing time. Additionally, an isothermal curing stage carried out at the resin’s lowest viscosity should be added before the prepreg laminate is fully cured at high temperatures, since fiber wetting at the first isothermal stage is beneficial for reducing the void content. On the other hand, the presence of voids within a CFRP composite would normally reduce mechanical performances [41]; microwave-cured composites exhibiting higher void contents but exhibiting comparable or even better mechanical properties might be a result of the better interfacial properties achieved with microwave-curing processes than with thermal curing processes. These improved interfacial properties may play more important roles than void contents in mechanical properties, and similar results have been reported previously for microwave-cured carbon fiber/epoxy composites [14,16].

## 4. Conclusions

In this paper, a novel microwave system equipped with a three-dimensional motion system was proposed for curing prepreg composite laminates. This study proposes a curing process for unidirectional laminates (dimensions of 300 mm × 300 mm × 2 mm) by using a feasible and reliable process, with the optimal curing process including the following: isothermal curing at 110 °C for 10 min followed by at 140 °C for 30 min; microwave power increased from 520 W to 680 W stepwise; and the prepreg laminate simultaneously performing progressive rotations and reciprocating linear movements during the entire curing process. The microwave system is equipped with a six-point fiber optical fluorescence measurement system to monitor the temperature distribution on the prepreg laminate when it is radiated by microwaves, and the best temperature deviation could be obtained within 8.7 °C. FT−IR analyses indicate that microwave heating would lead to slight differences in the moisture and methylene content in the cured samples. DMA analyses demonstrated that a multistep microwave-curing process carried out at 110 °C for 10 min followed by 120 °C for 120 min could yield the highest glass transition temperature at 115.5 °C. The optical microscopy and mechanical analyses indicate that the void content can be lowered to 4.7%, and the tensile strength and compressive strength increased by 20.3% and 63.2%, respectively, when the prepreg laminate was cured by optimal processes.

## Figures and Tables

**Table 1:** Various microwave cure cycles of composite laminates.

Cure cycle	Isothermal Curing Temperature and Curing Time
M1	120 °C for 120 min
M2	110 °C for 10 min + 120 °C for 120 min
M3	140 °C for 30 min
M4	110 °C for 10 min + 140 °C for 30 min

**Table 2:** DMA results for laminates prepared by various processes.

Cure Cycle	Storage Modulus at 30 °C (MPa)			DMA Tg (°C)		
	Coupon 1	Coupon 2	Average Value	Coupon 1	Coupon 2	Average Value
TC	45,029	39,797	42,413	106.2	108.8	107.5
M1	52,403	40,191	46,297	103.5	103.1	103.3
M2	39,703	38,320	39,012	115.5	115.4	115.5
M3	37,710	38,502	38,106	104.1	107.5	105.8
M4	59,329	55,624	57,477	113.1	112.1	112.6

**Table 3:** Physical and mechanical properties of composite panels.

Properties	TC	M1	M2	M3	M4
Maximum temperature deviation (°C)	N/A	12.1	13.8	8.7	10.3
Glass transition temperature (°C)	107.5	103.3	115.5	105.8	112.6
Void content (%)	8.4	11.4	9.9	15.5	4.7
Average tensile strength (MPa)	1696.21	1893.86	1364.17	1777.19	2039.91
Average tensile modulus (GPa)	121.16	139.82	115.32	148.14	147.81
Average compressive strength (MPa)	443.72	732.07	635.22	605.00	724.00
Average compressive modulus (GPa)	101.75	145.92	131.29	38.44	135.44

## Data Availability

The data can be provided by the corresponding author upon request.

## Supplementary Materials

The following supporting information can be downloaded at: https://www.mdpi.com/article/10.3390/ma16020705/s1, Figure S1: Rheologram of the carbon fiber epoxy prepreg in dynamic heating processes at a constant heating rate of 1 °C/min; Figure S2: The heat flow evolution in the isothermal scans at 120 °C and 140 °C. Figure S3: Temperature characteristics of (a) M1, (b) M2, and (c) M4 cycles.
