# Supplementary material for: Improvements in Temperature Uniformity in Carbon Fiber Composites during Microwave-Curing Processes via a Recently Developed Microwave Equipped with a Three-Dimensional Motion System
Source: Materials (Basel). 2023 Jan 11;16(2):705. doi: 10.3390/ma16020705 (PMC9864177; doi:10.3390/ma16020705)
Supplement: Supplementary file 1 [file materials-16-00705-s001.zip › materials-2078970-supplementary.pdf]

1. Rheological characterization

A rheological characterization of the uncured prepreg was carried out using a Brookfield RST-CPS rheometer. Disposable parallel plates with a diameter of 25mm were used throughout. The measurement was carried out in the dynamic mode at a frequency of 1 Hz with a deformation of 1%. The test was started from the room temperature and performed up to 200 °C at a heating rate of 1 °C/min. The viscoelastic properties of the sample during cure, including the complex dynamic viscosity (η*), shear storage modulus (G'), and shear loss modulus (G''), were monitored.

According to the rheogram of the unreacted prepreg (Fig. S1), the prepreg displays lowest complex viscosity when the temperature is increased to 110 °C. Therefore, an additional isothermal segment is carried at 110 °C for 10 min in M2 and M4 cycles, which is supposed to improve the fiber surface wetting by the resin matrix during this stage.

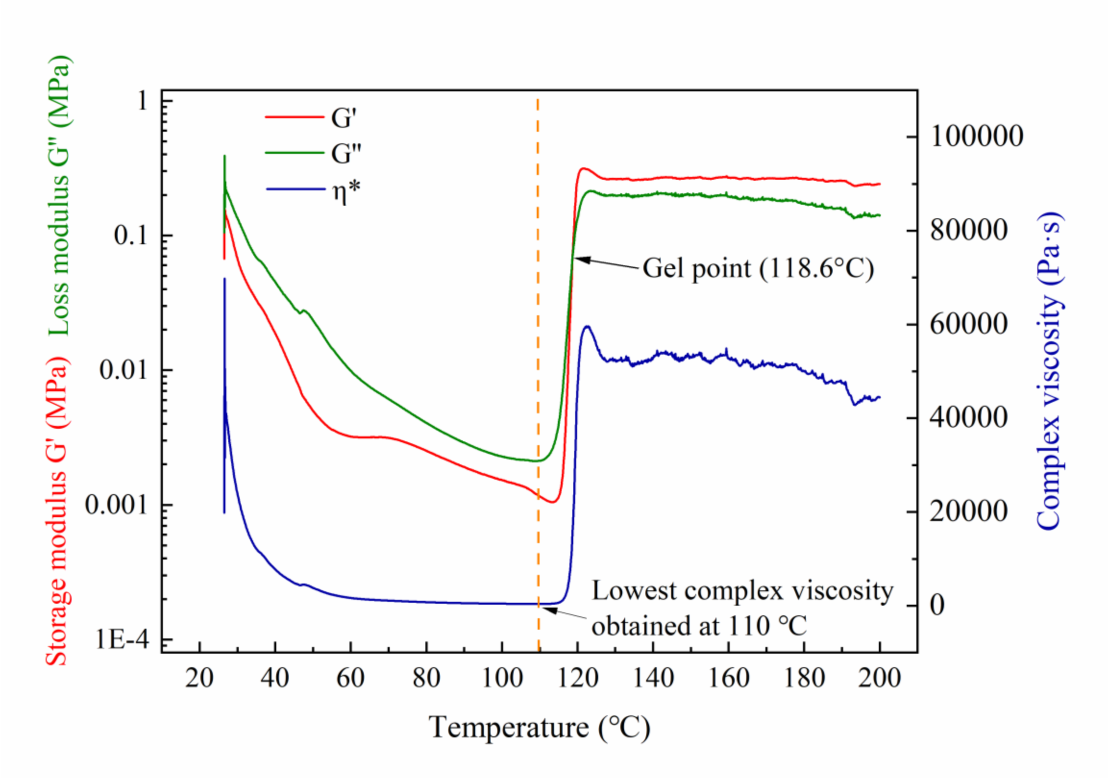

Figure S1. Rheologram of the carbon fiber epoxy prepreg in dynamic heating progresses at a constant heating rate of 1 °C/min.

2. Isothermal DSC scans

The differential scanning calorimetry (DSC) measurements were performed on a DSC 200F3 Netzsch, Germany. Samples were prepared by placing a small amount of uncured prepreg (9~10 mg) in an aluminum crucible, and the experiments were carried out under a constant flow of nitrogen of 50 ml/min. For the isothermal scans, the DSC chamber was immediately heated to the predetermined isothermal temperature at a heating rate of 50 °C/min, and held at that temperature for different periods of time until the DSC curve level off.

The isothermal scans obtained at 120 °C and 140 °C are shown in Fig. S2. As can be seen, the DSC curves level off after 55 min and 25 min at 120 °C and 140 °C, respectively.

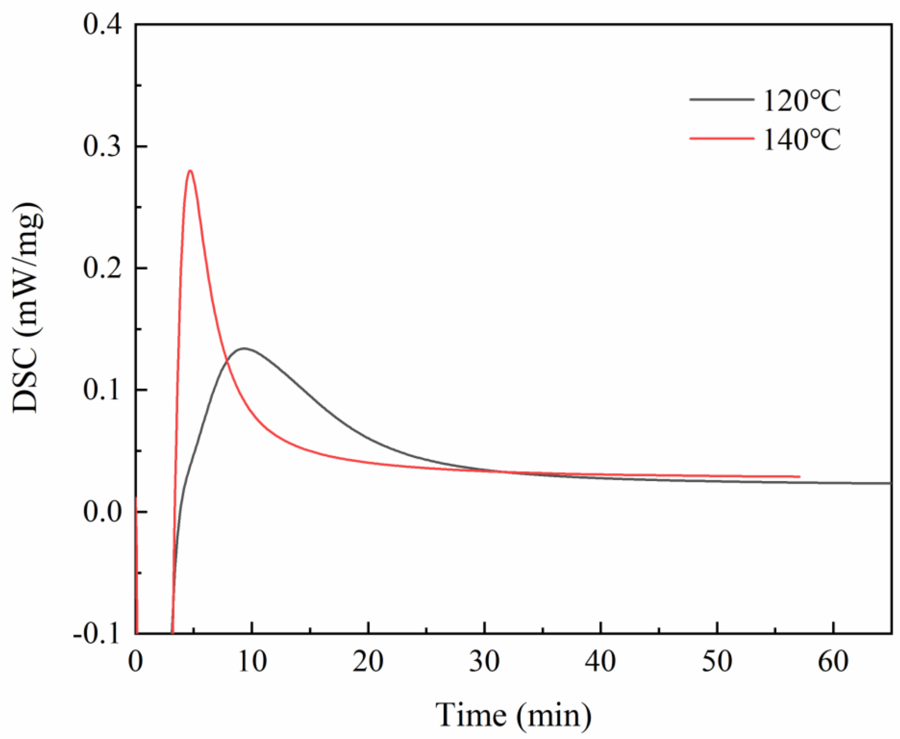

Figure S2. The heat flow evolution in the isothermal scans at 120 °C and 140 °C.

3. Microwave heating characteristics of various microwave curing cycles

The temperature curves of M1, M2 and M4 cycles during microwave curing processes are shown in Fig S3. It could be observed that the maximum temperature differences in these three cycles are 12.1 °C, 13.8 °C and 10.3 °C, respectively. As shown in Fig S3(a), in the M1 curing process, the point 6 fiber probe unfortunately fell down after the curing was carried out for ca. 52 min, resulting in the missing of monitoring of the temperature of point 6 in the left curing process. Moreover, it could be concluded that the temperature differences among six points would normally increase as the isothermal curing process undergoes compared with that of heating up segment, and prolonging the isothermal curing time is more likely to increase the temperature deviation. Therefore, a short curing time would be beneficial to controlling the temperature difference in microwave curing process.

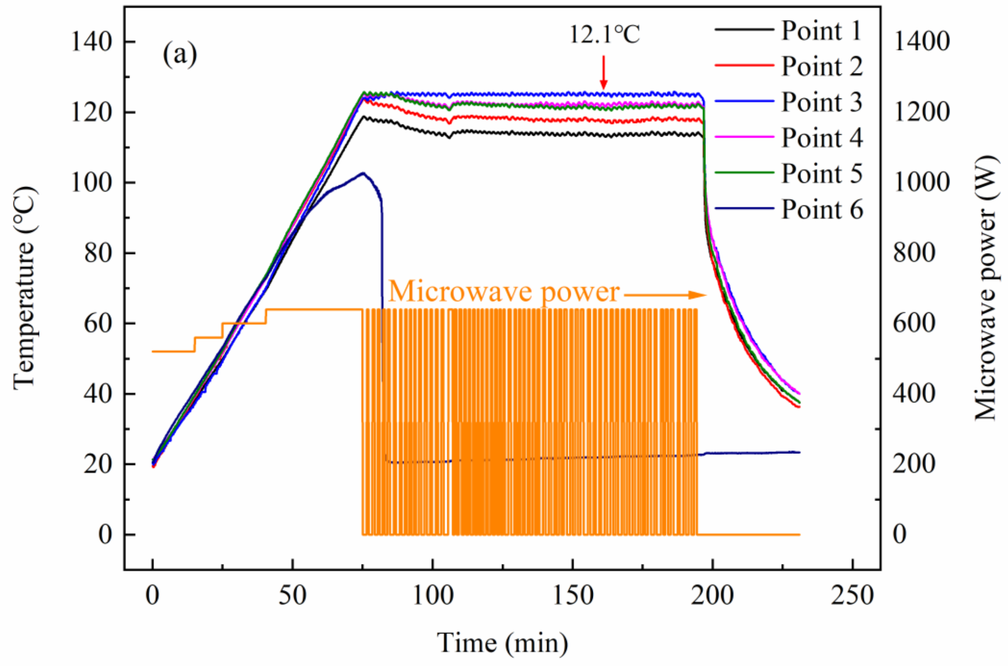

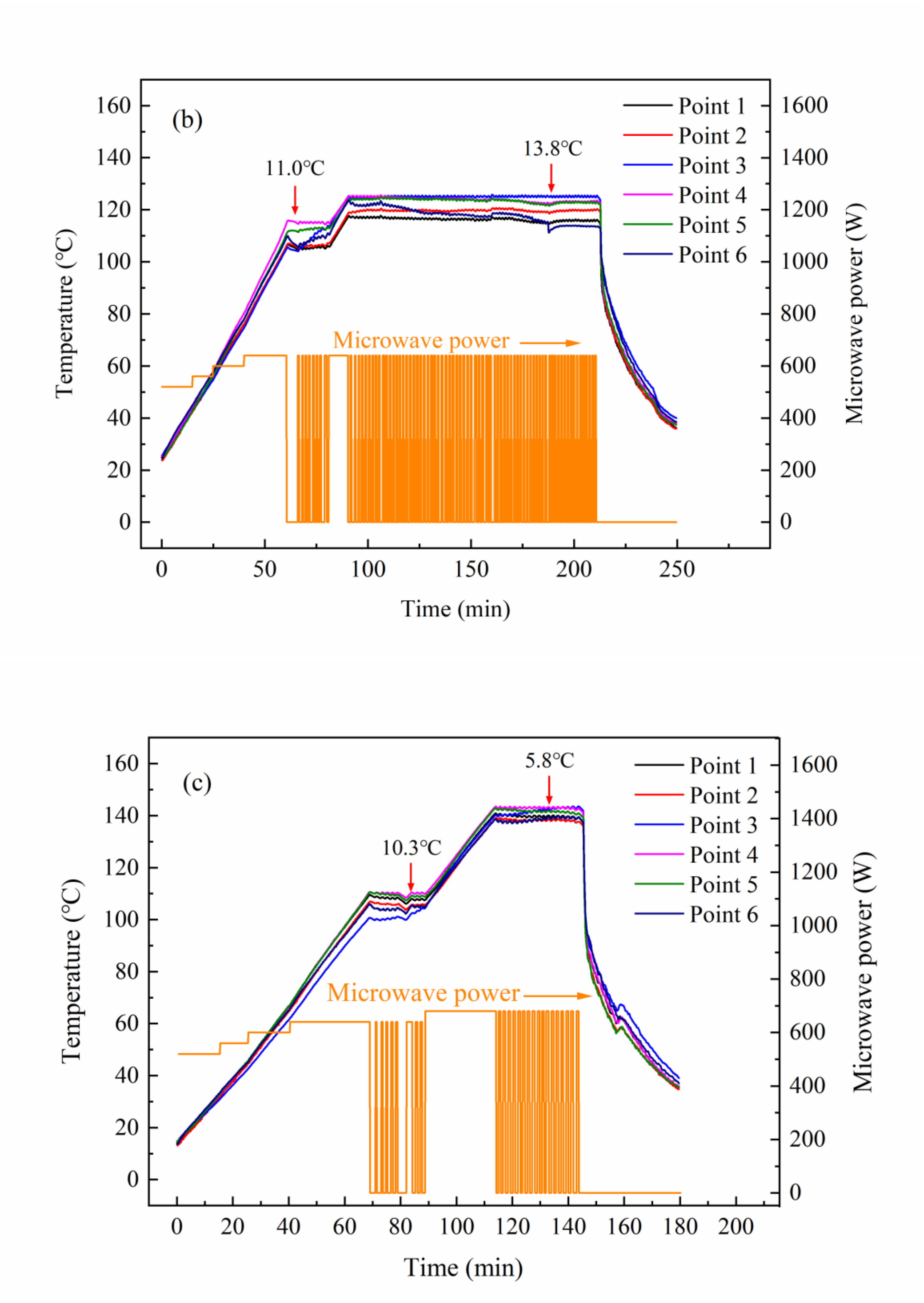

Figure S3. Temperature characteristics of (a) M1, (b) M2, and (c) M4 cycles.
